# How did consumers’ self-protective behavior formed during the COVID-19 pandemic?

**DOI:** 10.3389/fpsyg.2023.1075211

**Published:** 2023-03-09

**Authors:** Hu Xue, Shanshan Jin, Qianrong Wu, Xianhui Geng

**Affiliations:** College of Economics and Management, Nanjing Agricultural University, Nanjing, China

**Keywords:** COVID-19, protective action decision model, risk information, risk perception, self-protective behavior

## Abstract

**Introduction:**

This study explored the formation mechanism of consumers’ self-protective behavior during the COVID-19 pandemic, which is very important for policy settings to regulate consumer behavior. Based on the basic framework of the Protective Action Decision Model (PADM), this study analyzed the formation mechanism of consumers’ self-protective willingness from the perspective of risk information, and explained the deviation between consumers’ self-protective willingness and behavior from the perspective of protective behavior attributes.

**Methods:**

Based on 1,265 consumer survey data during the COVID-19 pandemic, the empirical test was carried out.

**Results and Discussion:**

The amount of risk information has a significant positive impact on the consumers’ self-protective willingness, where the credibility of risk information plays a positive moderating role between them. Risk perception plays a positive mediating role between the amount of risk information and the consumers’ self-protective willingness, and the positive mediating effect of risk perception is negatively moderated by the credibility of risk information. In the protective behavior attributes, hazard-related attributes play a positive moderating role between the consumers’ self-protective willingness and behavior, while resource-related attributes play the opposite role. Consumers pay more attention to hazard-related attributes than resource-related attributes, and they are willing to consume more resources to reduce risk.

## 1. Introduction

The human-to-human transmission characteristics (Chan et al., 2020; Li et al., 2020) of the COVID-19 create an extremely high-risk consumer environment. Consumers were required to wear masks with virus isolation function when they go out to buy daily necessities. However, masks cannot completely prevent consumers from exposure to the COVID-19 virus (Akhtar et al., 2020). Thus, there was still the risk that consumers were infected with the COVID-19 virus when they went out to buy daily consumables. Protection Motivation Theory considers that consumers have a protective motivation and adopt relevant self-protective behavior when they perceive the risk (Floyd et al., 2000). Self-protective behavior can restore the consumer to an initial state where there is no threat, danger, harm, or loss. To avoid being infected by the COVID-19 virus, consumers tended to adopt protective behavior such as online shopping (Hao et al., 2020; Youn et al., 2022), supermarket shopping (Li et al., 2020), stocking up on food (Wang et al., 2020; Scacchi et al., 2021), and panic buying (Prentice et al., 2020; Billore and Anisimova, 2021). The Chinese government’s prevention and control policy for the COVID-19 outbreak is to interrupt the transmission chain of the COVID-19 virus, and the key is to guide consumers to adopt low-risk behavior. If the government had guided consumers to adopt self-protective behaviors during the COVID-19 pandemic, and it effectively prevented the spread of the COVID-19 virus. This is also the reason why the COVID-19 pandemic in China has been quickly controlled compared with other countries that have suffered from the COVID-19 pandemic. This study examined the formation mechanism of consumers’ self-protective behavior, which aims to provide theoretical support and empirical evidence for policy settings to regulate consumer behavior during the COVID-19 pandemic.

Based on combing through the literature on consumer behavior during the public health events, the following three aspects were found to be worthy of attention. First, in most studies, scholars have indicated that risk perception is the main factor influencing consumers’ purchasing willingness (Myae and Goddard, 2020; Thomas and Feng, 2021). However, risk perception is not the initial factor influencing consumers’ purchasing willingness. In general, risk information is considered to be an important factor influencing the cognitive behavior of individuals, which triggers individual cognitive processes (Rogers, 1983). In the Protective Action Decision Model (PADM) proposed by Lindell and Perry (1992), risk information is the origination that individuals decide whether to adopt self-protective behaviors and is also an important factor in continuously adjusting an individual’s risk perception and the self-protective willingness. Thus, risk information plays a very important role in the evolution of risk perception and the self-protective willingness (Slater and Rasinski, 2005; Zavyalova et al., 2012). In summary, this study highlights the role of risk information in the formation process of the consumers’ self-protective willingness and the mediating role of risk perception between risk information and the consumers’ self-protective willingness, which helps us understand the formation mechanism of the consumers’ self-protective willingness and provides path support for enhancing the consumers’ self-protective willingness.

Secondly, consumers often adopt behaviors with high risk in real situations, such as eating risky foods (Xie et al., 2020), crowd gathering (Leung et al., 2020), and other behaviors. These phenomena may be caused by incomplete or incorrect dissemination of risk information (Cava et al., 2005; Lindell and Perry, 2012), i.e., incorrect and low credible risk information reduces the consumers’ self-protective willingness. This study argues that it is necessary to incorporate the credibility of risk information into the analytical framework.

Finally, a large number of studies in the existing literature argue that the individual’s actual behavior is determined through willingness. Ajzen (1991) proposed that willingness directly determined behavior when actual control conditions are sufficient, such as ability, resources, and opportunity. Fukukawa (2003) argued that willingness led directly to behavior in the consumer behavior decision process. However, as research has progressed, scholars have found that there is a deviation between the consumers’ self-protective willingness and behavior in the consumer behavior decision process (Auger and Devinney, 2007). In recent years, research on consumer behavior changes has increased significantly during the COVID-19 pandemic, but few studies have explored the factors that influence the deviation. This study will specifically examine the factors that contribute to the deviation between the consumers’ self-protective willingness and behavior.

This paper aims to evaluate the formation mechanism of consumers’ self-protective behavior during the COVID-19 pandemic. Thus, two research questions were formulated: (1) What factors can influence consumers’ self-protective willingness during the COVID-19 pandemic? What is the role of risk perception? (2) What factors influence the transformation of consumers’ self-protective willingness to behavior. This paper is original because it identifies a more complete formation mechanism of consumers’ self-protective behavior during the COVID-19 pandemic.

## 2. Theoretical framework and hypotheses

### 2.1. The protective action decision model

Lindell and Perry (1992, 2004, 2012) constructed and gradually improved the Protective Action Decision Model (PADM) by integrating theories of behavior decision-making, attitude-behavior relationships, and protective behavior. This model can identify factors that influence individuals to adopt protective behavior. The main purpose of the PADM is to analyze the decision-making process of individuals for protective action when experiencing environmental risks and hazards, and it is widely used for natural disasters (Terpstra and Lindell, 2013), environmental hazards (Zhu and Yao, 2018), public health events (Wang et al., 2018), and other types of risk events. The PADM consists of three sub-stages. First, environmental cues, social cues, and society warnings initiate the information-seeking stage, including exposure (do they receive the information), attention (do they notice the information), and comprehension (do they understand the information). Subsequently, the information-seeking triggers the risk perception stage of environmental threats, alternative protective actions, relevant stakeholders and other core perceptions. Finally, the cognitive process transitions into the protective behavior decision stage including a series of decisions such as risk identification, risk assessment, protective behavior search, protective behavior assessment, and protective behavior implementation.

Several studies have applied and tested the PADM to assess the self-protective willingness during the public health event outbreak (Wang et al., 2018; Johnson, 2019). While the PADM has been well validated in previous studies related to epidemics, it should be reexamined whether the theory applies to the unique risk environment created by the COVID-19 virus. Johnson and Mayorga (2021) used the variables of threat, protective behaviors, and stakeholder perceptions in the PADM to assess Americans’ early behavioral responses to COVID-19. Zheng et al. (2022) explored Internet users’ responses to health-related online fake news during the COVID-19 pandemic using the PADM. However, no studies have applied the PADM to assess consumer behavior during the COVID-19 pandemic.

Meanwhile, in contrast with the Transtheoretical Model of behavior Change (TTM) (Prochaska and Velicer, 1997) and the Precaution Adoption Process Model (PAPM) (Weinstein, 1988), protective behavior in the PADM is a temporary rather than permanent contingency action. The decision-making process for emergency response behavior can go through all stages of the PADM in a matter of minutes and is applicable to situations where emergency management personnel are simultaneously communicating information to a large number of people responding to a single crisis event, which is consistent with the reality of the COVID-19 pandemic in the context of this study. Although the PADM is more applicable to the realistic context of the COVID-19 pandemic than other protective behavior theories, it is still difficult to evaluate the complete formation mechanism of protective behavior based on a single theory. The PADM should be improved and extended by combining classical theories and the actual situation of the COVID-19 pandemic. Shi et al. (2021) explored the factors influencing protective behavior in China in the post-COVID-19 pandemic era based on the risk perception sentiment model and the PADM. El-Said and Aziz (2021) have integrated the Technology Acceptance Model (TAM) and the PADM to determine the factors that affect a person’s decision to adopt virtual tours as temporary alternatives during times of crisis.

This study extended the PADM that the protective behavior decision stage is divided into two parts: the self-protective willingness and self-protective behavior. The formation mechanism of the consumers’ self-protective willingness is analyzed by adding the credibility of risk information to the information-seeking stage. The transformation process of consumers’ self-protective willingness to behavior is carefully analyzed from the perspective of protective behavior attributes.

### 2.2. The formation mechanism of the consumers’ self-protective willingness during the COVID-19 epidemic

After the public health event, consumers’ risk perception fluctuates dramatically, and adopt various self-protective behaviors due to the uncertainty of the state evolution and the asymmetry of the epidemic information (Shapira et al., 2018). The higher the risk perception the more consumers tend to adopt self-protective behaviors (Feng et al., 2014), and tend to adopt self-protective behaviors that are effective and have lower resource-demanding (Terpstra and Lindell, 2013). The formation and evolution of risk perception depend on the source, coding, communication agent, communication channel, and frequency of risk information (Wei et al., 2012). By continuously seeking risk-related information, consumers’ risk perception may change dynamically with their selective understanding of the information, indicating that the formation and evolution of risk perceptions is a Bayesian learning process (Liu et al., 1998).

Lindell and Perry (1992) constructed the Protective Action Decision Model from the perspective of risk information flow and risk perception. The PADM is a continuous and multi-stage structure with feedback (Lindell and Perry, 2004). The process of PADM begins with information-seeking. This stage includes three sub-stages of warning exposure, attention, and understanding, and this stage is also a continuous and multi-stage structure with feedback. Consumers passively receive risk warning information and begin to pay attention and continuously understand it. This stage is the process of accumulating the amount of risk information. When the amount of risk information has reached a certain level, the process of PADM enters the risk perception stage. Similarly, there is a threshold value of consumers’ risk perception. If this threshold value is not reached, the process of PADM will return to the risk information seeking stage and continue to accumulate the amount of risk information to enhance the risk perception. When the risk perception exceeds this threshold, the process of PADM enters the protective behavior decision stage. In summary, the PADM is a conditional cyclic structure, that is, when uncertainty exists at a certain stage, risk information seeking occurs. Once the uncertainty is solved, it moves to the next stage of the process, and this continuous process does not stop running until the risk disappears, the protective behavior is adopted, or the protective behavior fails.

*Hypothesis* 1 (*H*1): The amount of risk information has a significant positive effect on the consumers’ self-protective willingness.

*Hypothesis* 2 (*H*2): Risk perception has a positive mediating effect between the amount of risk information and the consumers’ self-protective willingness.

The media plays a key role in the diffusion process of risk information and the formation of public risk perception (Wei et al., 2016). Situational Awareness Theory (SAT) considers that formal information sources (newspapers, press releases, and educational information) and informal information sources (social media, online reviews, family and peer perceptions) contribute to forming the public’s risk perception during the public health event outbreak (Qazi et al., 2020). However, there are significant differences in individuals’ information perception due to the use of different information collection channels, which in turn affects their risk perception and choice of protective behavior decisions. On the one hand, the amount of media coverage, overemphasis on threats, and the tone of coverage all may amplify the public’s risk perceptions (Klemm et al., 2016). Allington et al. (2020) studied the impact of media use on protective behaviors of UK residents during the COVID-19 pandemic and found that the use of unregulated social media (e.g., social media) as an information source posed a health risk to the public due to its potential to spread health-related conspiracy information. On the other hand, in the age of the Internet, especially self-media, the public is no longer simply a passive recipient of information but also acts as a producer and proliferator of information, reflecting the characteristic that everyone is a media platform (Vuoric and Okkonen, 2012). The public, as amateurs, may be unable or unwilling to fully analyze the risk information they receive (McComas, 2006). Studies of U.S. adults sharing information related to Novel Coronavirus Pneumonia have shown that people often share false statements about Novel Coronavirus Pneumonia, partly because they do not adequately consider the accuracy of the risk information when sharing it (Pennycook et al., 2020).

In summary, the effect of different information channels on risk perception and protective behavior adoption can be explained in two ways. On the one hand, appropriate, timely, data-based health information is important to increase the consumers’ self-protective willingness (Poland, 2010). On the other hand, the reputation of the organization plays an important role in risk communication. The reputation of the organization in the risk environment has a buffering protective effect (mitigating effect), which can mitigate the loss of organizational value after a disaster loss occurs (Wei et al., 2017). Dai et al. (2020) proposed a mediation model based on PADM to explain the effects of government interventions and individual factors on protective behaviors during the COVID-19 pandemic, and showed that government emergency public information such as detailed outbreak information and positive risk communication had a greater impact on protective measures than refuting rumors.

Risk information with high credibility is more easily received by individuals. In comparison, risk information with low credibility is difficult to receive, and individuals need to process this risk information more systematically (Trumbo and Mccomas, 2003). Therefore, the multi-stage process of PADM is difficult to continuously run in the reality of the situation. Due to risk information with high credibility being received in the information-seeking stage, the individual skips the risk perception stage and steps directly to the final protective behavior decision stage. An extremely credible (or powerful) source might obtain immediate and unquestioning adopt protective behavior, even if there were no explanation why protective behavior was necessary or what protective behavior was feasible (Gladwin et al., 2001). For example, residents in Wuhan were ordered to comply with home quarantine during the COVID-19 pandemic, and cannot go out to buy fresh food. Fresh food was centrally purchased by the communities and distributed to residents. While residents were likely to disobey the regulation if the regulated information came from unofficial media, residents would strictly follow the regulation if the regulated information came from official media.

*Hypothesis* 3 (*H*3): The credibility of risk information has a positive moderating effect between the amount of risk information and the consumers’ self-protective willingness.

*Hypothesis* 4 (*H*4): The positive mediating effect of risk perception is negatively regulated by the credibility of risk information. The higher the credibility of risk information, the smaller the positive mediating effect of risk perception.

### 2.3. The mechanism of the willingness of consumers’ self-protective behavior transforming into actual consumers’ self-protective behavior during the COVID-19 pandemic

There is an inconsistency between the willingness and behavior in the consumer behavior decision process, and observing this gap is important for explaining, predicting, and influencing consumer behavior (Bagozzi, 1993). However, this gap still has not been sufficiently understood so far, especially in consumer behavior research (Auger et al., 2003; Belk et al., 2005; De Pelsmacker et al., 2005). Although the theoretical framework underlying the TPB tended to accept the theoretical assumption that willingness directly determines actual behavior, it also pointed out that behavior was influenced not only by the willingness, but also by the personal abilities, opportunities, and resources of the individual performing the behavior. And these factors are known as perceived behavioral control. The perceived behavioral control is very similar to the concepts of self-efficacy and coping costs proposed by Protection Motivation Theory (PMT) (Floyd et al., 2000). In addition, the PMT also presented response efficacy, which is an assessment of whether protective behavior is effective in reducing risk. Reactive efficacy, self-efficacy, and coping costs were considered to be three attributes of protective behavior and were the basis of individuals’ protective behavior decisions. However, the measure of self-efficacy in PMT already covers the concept of coping costs, so self-efficacy is not as predictive as it should be in practical applications.

The PADM reorganized the protective behavior attributes, and Lindell and Perry (2012) identified two attributes of protective behavior, namely hazard-related attributes and resource-related attributes. Hazard-related attributes are measured from the following three aspects: First, whether the protective behavior can effectively protect physical health against the hazards. Second, whether the protective behavior can effectively protect property against the hazards. Third, whether the protective behavior can not only avoid the hazards but also benefit other aspects. The PADM considers that the protective behavior should not only protect individuals against hazards but also be beneficial in other ways, while PMT does not indicate this level of meaning. Thus, the concept of hazard-related attributes in PADM is more specific and detailed. Resource-related attributes refer to the requirement of adopting certain protective behaviors (Lindell et al., 2009). Although the concept of coping costs is indicated in the PMT, it only encompasses three aspects: time, money, and effort expended. The resource-related attributes in the PADM point out that costs include money, specialized skills, knowledge or tools, energy, time, and communication with others. Although the need for specialized skills, knowledge, or tools is similar to the concept of self-efficacy in the PMT, there is no doubt that the resource-related attributes in the PADM are more specific.

Previous studies using the PADM did not consider protective behavior attributes as influencing factors for the deviation between the self-protective willingness and behavior, but only as influencing factors for self-protective behavior. And there is no insight into the mechanism of protective behavior attributes. This study would explain the deviation between the consumers’ self-protective willingness and behavior from the perspective of protective behavior attributes. According to the PADM, protective behavior attribute is another core element of individuals’ behavior response to risk. Hazard-related attributes generally exhibit positive correlations with the self-protective willingness and behavior, whereas resource-related attributes are negatively correlated with the self-protective willingness an behavior (Lindell and Perry, 2004). Terpstra and Lindell (2013) believed that individuals may be more confident in performing protective behaviors when they perceive a higher level of hazard-related attributes, which promoted the transformation of willingness into actual behavior. When the risk proximity is very high, people actively adopt protective behaviors. Because the risk proximity magnifies the hazard-related attributes of protective behaviors, and adopting protective behaviors can greatly reduce the risk. For example, during the COVID-19 pandemic, high-risk groups tended to eat more fruits and vegetables, which could enhance the body’s immunity (Moreb et al., 2021). Meanwhile, individuals may overestimate the cost of implementing protective behaviors when they perceive a higher level of resource requirement, thereby reducing their confidence in making risk adjustments (Terpstra and Lindell, 2013).

*Hypothesis* 5 (*H*5): The consumers’ self-protective willingness has a positive effect on the consumers’ self-protective behavior.

*Hypothesis* 6 (*H*6): Protective behavior attributes has a moderating effect between the consumers’ self-protective willingness and behavior.

**Figure 1:**
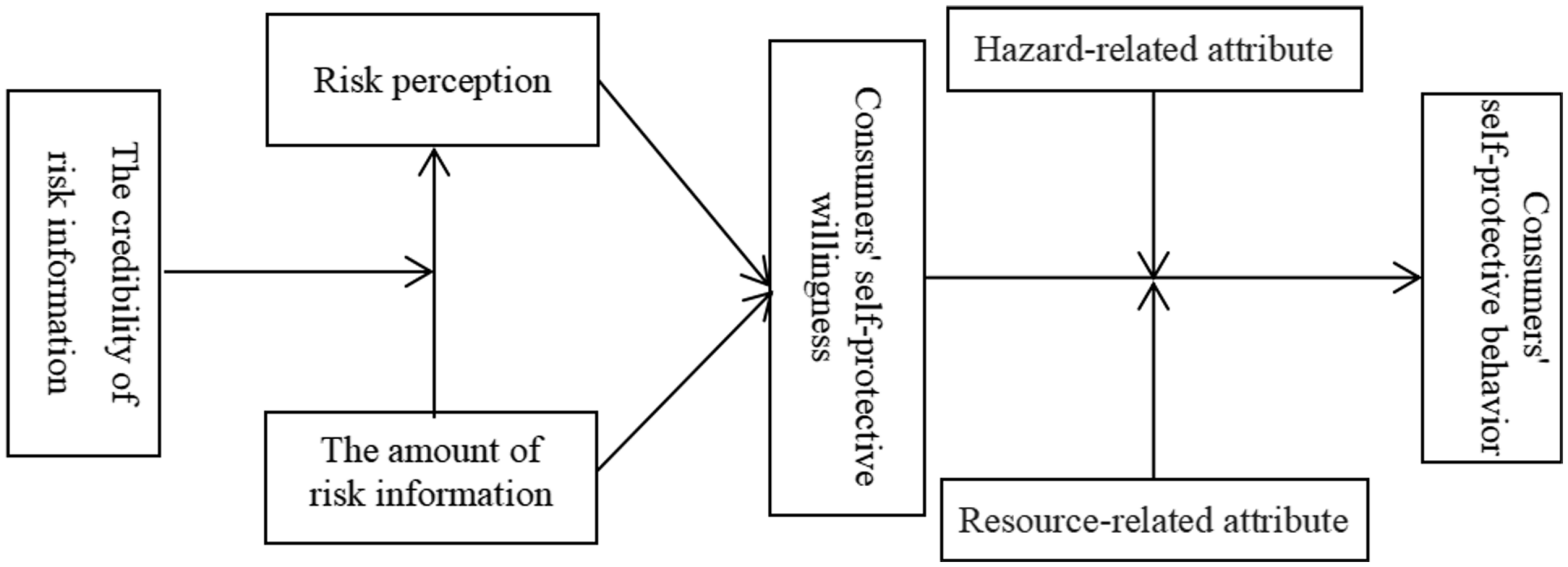
Research model.

Based on the theoretical derivation and hypotheses development, we constructed a research model (shown in Figure 1) and undertook an empirical test to explain the formation mechanism of consumers’ self-protective behavior during the COVID-19 pandemic.

## 3. Data and methodology

### 3.1. Sample and data collection

The Chinese government has adopted protective measures of home quarantine during the COVID-19 pandemic. Therefore, this study used an online survey for data collection. The collection period was from March 1 to March 15, 2020. This online survey collected 1,265 valid questionnaires. The survey respondents come from 30 provinces in China, except for Tibet, Hong Kong, Taiwan, and Macau. These questionnaires were collected by China’s professional marketing research company (SO JUMP). To be eligible for the survey, the respondents must be the main consumers in their households. Furthermore, the respondents should be at least 18 years old and above. In order to improve the quality of the data, we used the “trap question” to identify respondents who did not read the survey questions carefully and deleted them from the sample set. ^1^ The questionnaires in this study were open to all internet users. One hundred thirty-five invalid questionnaires were eliminated through the ‘trap question’, and the valid questionnaire rate was 90.4%.

The demographic profiles of the respondents, such as gender, age, educational level, and household income, are summarized in Table 1. Approximately 54.78% of the respondents are female. In terms of living location, approximately 63.72% of the respondents lived in city, approximately 23.32% of respondents lived in town, and approximately 12.96% of respondents lived in the village. In terms of age, approximately 30.04% of the respondents were aged between 26 and 30 years, and approximately 41.66% of the respondents aged between 31 and 40 year. In terms of monthly household income, approximately 16.13% of the respondents’ monthly household income ranged between 9,001 and 11,000 yuan, and approximately 20.32% of the monthly household income ranged between 11,001 and 15,000 yuan. In terms of education level, approximately 73.52% of the respondents had a university degree (undergraduate or graduate).

**Table 1:** Demographic profile of respondents (*n* = 1,265).

Variable	Measure	Frequency	Percentage (%)
Gender	Male	572	45.22
	Female	693	54.78
Location	City	806	63.72
	Town	295	23.32
	Village	164	12.96
Age	18–25	175	13.83
	26–30	380	30.04
	31–40	527	41.66
	41–50	134	10.59
	More than 50	49	3.87
Income	Less than ￥5,001	96	7.59
	￥5,001–￥7,000	188	14.86
	￥7,001–￥9,000	175	13.83
	￥9,001–￥11,000	204	16.13
	￥11,001–￥15,000	257	20.32
	￥15,001–￥20,000	185	14.62
	Over ￥20,000	161	12.73
Education	Less than high school	13	1.03
	High school	78	6.17
	Vocational school	217	17.15
	College graduate	825	65.22
	Masters’ degree or PhD	105	8.30

### 3.2. Measurement

To ensure the validity of the variable measurement method, we have adopted the scale used in previous studies as much as possible. All items were rated on a 5-point Likert scale ranging (shown in Table 2).

**Table 2:** Key variables and questionnaire items.

Variables	Source	Items	Measure
Risk information	Zhu and Yao (2018), Hu et al. (2019)	*Official-media:* How much information about the COVID-19 pandemic do you get from official-media during the COVID-19 pandemic? *Self-media:* How much information about the COVID-19 pandemic do you get from self-media during the COVID-19 pandemic? *Informal source:* How much information about the COVID-19 pandemic do you get from informal source during the COVID-19 pandemic?	Not at all = 1, small extent = 2, moderate extent = 3, great extent = 4, very great extent = 5
Risk perception of COVID-19	Sapp and Downing-Matibag (2009)	*RP 1:* I think that there is a high probability that I am infected with the COVID-19 virus. *RP 2:* I think that I will die when I am infected with the COVID-19 virus. *RP 3:* I think that people around me (family and friends) will be infected by the COVID-19 virus when I am infected.	Strongly disagree = 1, disagree = 2, neutral/moderate = 3, agree = 4, strongly agree = 5
Consumers’ self-protective willingness	Durham et al. (2012)	What percentage of daily necessities were you willing to purchase through supermarkets during the COVID-19 pandemic?	0–20% = 1, 21–40% = 2, 41–60% = 3, 61–80% = 4, 81–100% = 5
Consumers’ self-protective behavior	Durham et al. (2012)	What percentage of daily necessities did you purchase through supermarkets during the COVID-19 pandemic?	0–20% = 1, 21–40% = 2, 41–60% = 3, 61–80% = 4, 81–100% = 5
Hazard-related attributes	Lindell et al. (2016)	I think that it will help me to reduce my risk by shopping at the supermarket during the COVID-19 pandemic. First it is effectively preventing the respondent from being infected by the virus, and second it is effectively avoiding the risk of transmitting the virus to other people.	Strongly disagree = 1, disagree = 2, neutral/moderate = 3, agree = 4, strongly agree = 5
Resource-related attributes	Lindell et al. (2016)	I think that shopping through supermarkets consumes a lot of resources during the COVID-19 pandemic, such as money, effort or time, specialized knowledge, cooperation from others.	Strongly disagree = 1, disagree = 2, neutral/moderate = 3, agree = 4, strongly agree = 5

Information attention refers to the extent of respondents’ reliance on certain information sources to obtain risk information (Zhu and Yao, 2018). This study selected two aspects of the amount of information and the credibility of information to measure information attention. Drawing on the experience of China’s past pandemics, traditional mass media (such as television, radio, and newspapers) are the main source of pandemic information disseminated by the Chinese government (Vong et al., 2014) and the main source of risk information for Chinese residents (Wei et al., 2010, 2012). The reason is that it is easily accessible and has a high response rate and vivid descriptions. It can be identified as the official source. However, with the rapid development of information technology, the traditional mass media have evolved into an all-media era, in which social media has become an important part of the risk information transmission system (Hu et al., 2019), but risk information with low credibility transmitted by social media will have a serious impact (Hirose and Sonehara, 2008). Therefore, we divided official sources into official-media and self-media. In addition, risk information transmitted by casual interpersonal interactions between friends constitutes an ‘informal source’ (Zhu et al., 2011). The credibility of information sources follows the following growth pattern: informal sources, self-media, and official-media.

The measurement indicators of the risk perception of COVID-19 were adapted from the scale of Peltz et al. (2007) and Sapp and Downing-Matibag (2009). The reliability of the risk perception of COVID-19 was adequately indicated by the Cronbach’s alpha of the construct (Cronbach’s alpha = 0.788).

This study chose supermarket shopping as the research subject. Generally speaking, Chinese consumers can buy daily necessities from e-commerce, farmers’ markets and supermarkets. During the COVID-19 pandemic, Chinese consumers were not able to buy daily necessities from e-commerce due to the government’s home quarantine policy and traffic control policy, and Chinese consumers had to choose between farmers’ markets and supermarkets to buy daily necessities. However, the pandemic of COVID-19 broke out in the farmers’ markets of Wuhan, and the cluttered nature of the farmers’ markets create an extremely high-risk consumer environment. The supermarkets provide wipes for cleaning trolleys/basket handles and hand sanitizers, which are placed around the supermarkets. Consumers have to pack their own bags and have fewer checkout operations so that the consumers maintain a physical distance from the staff. Therefore compared to buying daily necessities at farmers’ markets, it is safer to buy daily necessities at supermarkets to avoid being infected by the virus. We asked the respondents to report their willingness and actual situation of supermarket shopping.

The measurement indicators of hazard-related attributes and resource-related attributes were adapted from the scale of Lindell et al. (2016). We asked the respondents to report their scores on hazard-related attributes and resource-related attributes of supermarket shopping. Among them, supermarket shopping exhibits two hazard-related attributes. First it is effectively preventing the respondent from being infected by the virus, and second it is effectively avoiding the risk of transmitting the virus to other people. According to Lindell et al. (2016), four resource-related attributes are the amount of money, the requirement of specialized knowledge and skills, the requirement of considerable effort, and the requirement of deep cooperation with other individuals. The measurement of resource-related attributes was based on the rating of respondents on the extent of required resources when taking supermarket shopping.

### 3.3. Empirical models

In terms of estimation methods, since variables such as the consumers’ self-protective willingness and behavior are measured by ordered discrete variables, the *ordered probit* estimation or *ordered logit* estimation should be used. However, Ferrer-I-Carbonell and Frijters (2004) found that the sign and significance of the regression coefficients obtained by using *ordered probit* or *ordered logit* estimation are consistent with those obtained by using *ordinary least squares* (OLS) estimation. Many studies directly used *ordinary least squares* (OLS) estimation to deal with ordered choice variable models (Brockmann et al., 2009; John et al., 2009; Jiang et al., 2012). At the same time, considering that the results of *ordinary least squares* (OLS) estimation are more intuitive and easier to interpret, the *ordinary least squares* (OLS) estimation is also used in this paper.

Following previous studies (Ren et al., 2019; Shi et al., 2020), the Baron and Kenny’s approach is used to examine the relationship between risk information, risk perception, and the consumers’ self-protective willingness during the COVID-19 pandemic as well as investigate the potential mechanism (Baron and Kenny, 1986). The model can be given as:


(1) $$WP_{i}=\alpha_{0}+\alpha_{1}\times RI_{i}+\sum \alpha_{j+1}\times Control_{j,i}+u_{i}$$

(2) $$RP_{i}=\beta_{0}+\beta_{1}\times RI_{i}+\sum \beta_{j+1}\times Control_{j,i}+u_{i}^{\prime }$$

(3) $$WP_{i}=\omega_{0}+\omega_{1}\times RI_{i}+\omega_{2}\times RP_{i}+\sum \omega_{j+2}\times Control_{j,i}+u_{i}^{\prime \prime }$$

where *RI_i_* and *RP_i_* respectively represent the amount of risk information and the risk perception of the *i*th consumer. *WP_i_* denotes the self-protective willingness of the *i*th consumer. *Control_i_* is a vector of control variables that may affect the willingness of the *i*th consumer. $u_{i}$, $u_{i}^{\prime }$ and $u_{i}^{\prime \prime }$ are error terms.

In order to test the possible influencing factors of the deviation between the consumers’ self-protective willingness and behavior, we believe that the protective behavior attributes is the main influencing factor. Therefore, we construct an econometric model for empirical testing. The model can be expressed as:


(4) $$\begin{array}{l} BP_{i}=\varphi_{0}+\varphi_{1}\times WP_{i}+\varphi_{2}\times WP_{i}\times HA_{i}+\varphi_{3}\times WP_{i}\\ \;\;\;\;\;\;\;\;\;\;\;\;\;\;\times RA_{i}+\sum \varphi_{j+3}\times Control_{j,i}+\mu_{i} \end{array}$$

where *HA_i_* and *RA_i_*, respectively, represent hazard-related attributes and resource-related attributes of the *i*th consumer. *BP_i_* denotes the self-protective behavior of the *i*th consumer. $\mu_{i}$ is error terms.

## 4. Results

### 4.1. The impact of risk information on the consumers’ self-protective willingness

The results of eq. (1) estimated by *ordinary least squares* (OLS) are reported in Model 1, Model 4, and Model 7 of Table 3. The results show that the amount of risk information from three information sources has significantly increased the consumers’ self-protective willingness. Consumers focusing on the risk information tend to have a higher consumers’ self-protective willingness. Therefore, it provides support for H1. At the same time, the estimated coefficient of the amount of risk information from the official-media is the largest, followed by the estimated coefficient of the amount of risk information from the self-media, and the estimated coefficient of the amount of risk information from informal resource is the smallest, which indicates that the credibility of risk information has a positive moderating effect in the positive influence of the amount of risk information on the consumers’ self-protective willingness. That is, the higher the credibility of risk information, the higher the influence of the amount of risk information on the consumers’ self-protective willingness. Therefore, it provides support for H3.

**Table 3:** The formation mechanism of the consumers’ self-protective willingness during the COVID-19 pandemic.

	Official-media			Self-media			Informal source		
	Willingness	Risk perception	Willingness	Willingness	Risk perception	Willingness	Willingness	Risk perception	Willingness
	Model 1	Model2	Model 3	Model 4	Model 5	Model 6	Model 7	Model 8	Model 9
Risk perception			0.2750*** (0.0343)			0.2725*** (0.0346)			0.2800*** (0.0345)
Risk information	0.1413*** (0.0355)	0.0778*** (0.0285)	0.1199*** (0.0348)	0.0883*** (0.0261)	0.1108*** (0.0208)	0.0581** (0.0258)	0.0579* (0.0300)	0.0995*** (0.0239)	0.0300 (0.0294)
Gender	0.0086 (0.0568)	0.0809* (0.0456)	−0.0137 (0.0555)	0.0129 (0.0570)	0.0870* (0.0453)	−0.0108 (0.0557)	0.0110 (0.0571)	0.0862* (0.0455)	−0.0131 (0.0558)
Age	0.0266 (0.0294)	−0.0379 (0.0236)	0.0370 (0.0287)	0.0324 (0.0295)	−0.0308 (0.0234)	0.0408 (0.0288)	0.0292 (0.0295)	−0.0337 (0.0235)	0.0387 (0.0288)
Education	0.0694* (0.0413)	−0.0424 (0.0332)	0.0811** (0.0403)	0.0680 (0.0414)	−0.0440 (0.0329)	0.0800*** (0.0405)	0.0695* (0.0415)	−0.0419 (0.0331)	0.0813** (0.0405)
Income	0.0786*** (0.0170)	0.0331** (0.0136)	0.0695*** (0.0166)	0.0830*** (0.0170)	0.0381*** (0.0135)	0.0726*** (0.0167)	0.0808*** (0.0171)	0.0362*** (0.0136)	0.0707*** (0.0167)
Location	−0.1157*** (0.0283)	0.0212 (0.0228)	−0.1216*** (0.0277)	−0.1200*** (0.0284)	0.0156 (0.0226)	−0.1243*** (0.0278)	−0.1174*** (0.0285)	0.0179 (0.0227)	−0.1224*** (0.0278)
Cons	2.7512*** (0.2632)	3.2167*** (0.2114)	1.8664*** (0.2795)	3.0423*** (0.2353)	3.1697*** (0.1871)	2.1785*** (0.2547)	3.1565*** (0.2383)	3.2293*** (0.1897)	2.2523*** (0.2578)
*R*-squared	0.0654	0.0151	0.1110	0.0621	0.0312	0.1062	0.0564	0.0228	0.1033
*N*	1,265	1,265	1,265	1,265	1,265	1,265	1,265	1,265	1,265

***, **, * represents significant at 1, 5, and 10% levels respectively, with standard errors in brackets.

### 4.2. The impact of risk information on risk perception

The results of eq. (2) estimated by *ordinary least squares* (OLS) are reported in Model 2, Model 5, and Model 8 of Table 3. The results show that the amount of risk information from three information sources has significantly increased the risk perception of COVID-19. At the same time, the estimated coefficient of the amount of risk information from the official-media is the largest, followed by the estimated coefficient of the amount of risk information from the self-media, and the estimated coefficient of the amount of risk information from informal resource is the smallest, which indicates that the credibility of risk information has a negative moderating effect in the positive influence of the amount of risk information on the risk perception of COVID-19. That is, the higher the credibility of risk information, the higher the influence of the amount of risk information on the risk perception of COVID-19.

### 4.3. The mediation effect of risk perception

The results of eq. (3) estimated by *ordinary least squares* (OLS) are reported in Model 3, Model 6, and Model 9 of Table 3. The results show that the risk perception of COVID-19 has significantly increased the consumers’ self-protective willingness. By comparing the estimated coefficient of the amount of risk information in eqs (1) and (3), this study finds that if risk perception as the mediator variable is further controlled, the estimated coefficient of the amount of risk information decrease. Among them, the estimated coefficient of the amount of risk information from the official-media has changed from 0.1413 to 0.1199, the estimated coefficient of the amount of risk information from the self-media has changed from 0.0883 to 0.0581, and the estimated coefficient of the amount of risk information from informal resource has changed from 0.0579 to 0.03, indicating that risk perception of COVID-19 has a positive mediating effect on the influence of the amount of risk information on the consumers’ self-protective willingness. Therefore, it provides support for H2.

The results in Table 3 also calculate the relative values of direct effect and indirect effect. That is the ratio of the direct effect and the indirect effect to the total effect. In the three information source models, the relative value of the indirect effect of the risk perception of COVID-19 in descending order is official-media (15.1%), self-media (34.2%), and informal resource (100%), indicating that the positive mediating effect of COVID-19 risk perception is regulated by the credibility of risk information. That is, the higher the credibility of risk information, the smaller the mediating effect of COVID-19 risk perception, the higher the direct effect of risk information on the consumers’ self-protective willingness. Therefore, it provides support for H4.

### 4.4. The mechanism of the consumers’ self-protective willingness transforming into the consumers’ self-protective behavior during the COVID-19 pandemic

Table 4 reports the estimation results for eq. (4), showing the relationships between the protective behavior attributes and the consumers’ self-protective behavior during the COVID-19 pandemic. It also shows the influence of the protective behavior attributes on the deviation between the consumers’ self-protective willingness and behavior.

**Table 4:** The mechanism of the consumers’ self-protective willingness transforming into the consumers’ self-protective behavior during the COVID-19 pandemic.

	Model 1
Willingness	0.5198*** (0.0840)
Willingness × Hazard-related attributes	0.0771*** (0.0131)
Willingness × Resource-related attributes	−0.0284*** (0.0082)
Gender	0.2426*** (0.0760)
Age	0.0324 (0.0393)
Education	0.0768 (0.0553)
Income	0.1107*** (0.0230)
Location	−0.1689*** (0.0381)
Cons	−2.1249*** (0.3270)
*R*-squared	0.3823
*N*	1,265

***, **, * represents significant at 1, 5, and 10% levels respectively, with standard errors in brackets.

The results of eq. (4) estimated by *ordinary least squares* (OLS) are reported in Table 4. The results show that the consumers’ self-protective willingness has significantly promoted the consumers’ self-protective behavior. Therefore, it provides support for H5. The results show that hazard-related attributes have significantly reduced the deviation between the consumers’ self-protective willingness and behavior, while resource-related attributes play the opposite role. Therefore, it provides support for H6. By comparing the regression coefficients of Willingness × Hazard-related attributes and Willingness × Resource-related attributes, it is found that the absolute values of the estimated coefficients of the Willingness × Hazard-related attributes are all greater than that of the Willingness × Resource-related attributes, indicating that consumers pay more attention to hazard-related attributes than resource-related attributes during the COVID-19 pandemic, and are willing to consume more resources to reduce risk.

## 5. Discussion

This study examines the formation mechanism of consumers’ self-protective behavior, which aims to provide theoretical support and empirical evidence for policy settings to regulate consumer behavior during the COVID-19 pandemic.

First of all, in most studies, scholars have indicated that risk perception is the main factor influencing consumers’ purchasing willingness (Myae and Goddard, 2020; Thomas and Feng, 2021). However, risk perception is not the initial factor influencing consumers’ purchasing willingness. In general, risk information is considered to be an important factor influencing the cognitive behavior of individuals, which triggers individual cognitive processes (Rogers, 1983). The finding from this research shows that the amount of risk information during the COVID-19 pandemic has a significant positive impact on the consumers’ self-protective willingness. That is, the more risk information about the COVID-19 pandemic is obtained, the stronger the consumers’ self-protective willingness will be. This result is supported by three different risk information source models. Therefore, to promote consumers to actively adopt consumers’ self-protective behaviors, governments, and other organizations need to convey risk information about the COVID-19 pandemic to consumers.

Secondly, there are significant differences in individuals’ perceptions of information obtained from different sources, which affect their risk perception and choice of self-protective behavior decisions (Qazi et al., 2020). Risk information with high credibility is more easily received by individuals. In comparison, risk information with low credibility is difficult to receive, and individuals need to process this risk information more systematically (Trumbo and Mccomas, 2003). The finding from this research shows that the credibility of risk information strengthens the relationship between the amount of risk information and the consumers’ self-protective willingness, which indicates that the higher the credibility of risk information is, the stronger the consumers’ self-protective willingness will be. In order to increase the consumers’ self-protective willingness during the pandemic, an indispensable path is to increase risk perception, and increasing the amount of risk information and the credibility of risk information are effective measures. Compared with the amount of risk information, the credibility of risk information is the focus of government in the Internet era. The government should especially monitor the credibility on self-media information in social media so that consumers can actively adopt protective behaviors at the beginning of the pandemic.

Finally, there is an inconsistency between the willingness and behavior in the consumer behavior decision process, and observing this gap is important for explaining, predicting, and influencing consumer behavior (Bagozzi, 1993). However, this gap still has not been sufficiently understood so far, especially in consumer behavior research (Auger et al., 2003; Belk et al., 2005; De Pelsmacker et al., 2005). The finding from this research shows that there is a certain deviation between the consumers’ self-protective behavior willingness and behavior. The hazard-related attributes will reduce this deviation, while resource-related attributes will increase this deviation. This finding shows that consumers’ self-protective behaviors are hindered by various practical conditions. For example, using e-commerce platforms to purchase food has a very low risk of being infected by the COVID-19 virus, because there is no contact with other people during the purchasing process. But the implementation of quarantine policies has caused huge problems in logistics services, commodity services, after-sales services, and commodity prices. That made using e-commerce platforms to purchase food difficult. Therefore, the government must make recommendations for relevant consumers’ self-protective behaviors and evaluate the barriers to implementing these consumers’ self-protective behaviors to ensure that these barriers can be eliminated during the pandemic.

## 6. Conclusion

This study explored the formation mechanism of consumers’ self-protective behavior during the COVID-19 pandemic, which is very important for policy settings to regulate consumer behavior. Based on the basic framework of the Protective Action Decision Model (PADM), this study analyzed the formation mechanism of consumers’ self-protective willingness from the perspective of risk information, and explained the deviation between consumers’ self-protective willingness and behavior from the perspective of protective behavior attributes. Based on 1,265 consumer survey data during the COVID-19 pandemic, the empirical test was carried out.

The results show that the amount of risk information has a significant positive impact on the consumers’ self-protective willingness, where the credibility of risk information plays a positive moderating role between them. Risk perception plays a positive mediating role between the amount of risk information and the consumers’ self-protective willingness, and the positive mediating effect of risk perception is negatively moderated by the credibility of risk information. In the protective behavior attributes, hazard-related attributes play a positive moderating role between the consumers’ self-protective willingness and behavior, while resource-related attributes play the opposite role. Consumers pay more attention to hazard-related attributes than resource-related attributes, and they are willing to consume more resources to reduce risk.

The present research provides several theoretical contributions to the consumer behavior literature and the protective behavior theory. The application of PADM in consumer research context is novel in the literature. To the best of our knowledge, this study is the first to apply the PADM to assess consumer behavior during the COVID-19 pandemic. Meanwhile, the extension of the PADM in this study is the latest work available. Secondly, this study advances the knowledge on the formation mechanism of consumers’ self-protective willingness. Different from previous studies, we used risk information as an initial factor that influences consumers’ self-protective willingness during the COVID-19 pandemic. Meanwhile, this research provides a more in-depth understanding of the impact of risk information on the consumers’ self-protective willingness by examining the effect of the amount of risk information and the credibility of risk information on the consumers’ self-protective willingness. Finally, this study explains the deviation between consumers’ self-protective willingness and behavior from the perspective of protective behavior attributes, thus promoting the existing literature. Meanwhile, this is a further extension of the PADM.

Like any research, this study has some limitations that also provide future research opportunities. Firstly, the study was implemented as a cross-sectional study after the declaration of COVID-19 as a global pandemic, but consumers’ self-protective behavior outcomes may need to be observed from a longitudinal perspective to understand the trends and changes in self-protective behavior. It would be meaningful to investigate post-COVID-19 consumers’ self-protective behavior and compare the results to those of the current study. Secondly, although the sample was diverse throughout the country and encompassed different age and education groups, the convenience sampling method was used. However, the results are applicable only to those consumers faced with the ‘lockdown’ and the pandemic purchasing experience, and therefore can be generalized to the Chinese population to a certain extent. Finally, Since the COVID-19 pandemic’s effects on consumers’ self-protective behavior vary in different countries because of different measures. Hence, a comparative, cross-national study could be conducted to provide a more holistic picture and greater insight into self-protective behavior.

## Data availability statement

The raw data supporting the conclusions of this article will be made available by the authors, without undue reservation.

## Author contributions

HX and XG were responsible for writing and contributed to the conception of the work, data analysis and interpretation, and drafting the article. SJ and QW contributed to the data collection, data analysis, and interpretation, and give important revising suggestions. All authors contributed to the article and approved the submitted version.

## Funding

This work was supported by China Agriculture Research System of MOF and MARA [Grant No. CARS-28].

## Conflict of interest

The authors declare that the research was conducted in the absence of any commercial or financial relationships that could be construed as a potential conflict of interest.

## Publisher’s note

All claims expressed in this article are solely those of the authors and do not necessarily represent those of their affiliated organizations, or those of the publisher, the editors and the reviewers. Any product that may be evaluated in this article, or claim that may be made by its manufacturer, is not guaranteed or endorsed by the publisher.

## Footnotes

^1^In the questionnaire, the question of whether the respondent is the main consumer in the household is set to ensure that the respondent has the thought process of consumer behavior and actual consumer behavior. At the same time, if there is a logical contradiction between the results of each question, the surveyed person’s questionnaire will be directly eliminated.

## References

[ref1] Ajzen I. (1991). The theory of planned behavior. Organ Behav. Hum. 50, 179–211. doi: 10.1016/0749-5978(91)90020-T

[ref2] Akhtar J. Garcia A. L. Saenz L. Kuravi S. Kota K. (2020). Can face masks offer protection from airborne sneeze and cough droplets in close-up, face-to-face human interactions? A quantitative study. Phys. Fluids 32:127112. doi: 10.1063/5.0035072, PMID: 33362404 PMC7757609

[ref3] Allington D. Duffy B. Wessely S. Dhavan N. Rubin J. (2020). Health-protective behavior, social media usage, and conspiracy belief during the COVID-19 public health emergency. Psychol. Med. 51, 1763–1769. doi: 10.1017/S003329172000224X, PMID: 32513320 PMC7298098

[ref4] Auger P. Burke P. Devinney T. M. Louviere J. J. (2003). What will consumers pay for social product features? J. Bus. Ethics 42, 281–304. doi: 10.1023/A:1022212816261

[ref5] Auger P. Devinney T. M. (2007). Do what consumers say matter? The misalignment of preferences with unconstrained ethical intentions. J. Bus. Ethics 76, 361–383. doi: 10.1007/s10551-006-9287-y

[ref6] Bagozzi R. P. (1993). On the neglect of volition in consumer research: a critique and proposal. Psychol. Market. 10, 215–237. doi: 10.1002/mar.4220100305

[ref7] Baron R. M. Kenny D. A. (1986). The moderator-mediator variable distinction in social psychological research: conceptual, strategic, and statistical considerations. J. Pers. Soc. Psychol. 51, 1173–1182. doi: 10.1037/0022-3514.51.6.1173, PMID: 3806354

[ref8] Belk R. Devinney T. M. Eckhardt G. (2005). Consumer ethics across cultures. Consum. Mark. Cult. 8, 275–289. doi: 10.1080/10253860500160411

[ref9] Billore S. Anisimova T. (2021). Panic buying research: a systematic literature review and future research agenda. Int. J. Consum. Stud. 45, 777–804. doi: 10.1111/ijcs.12669

[ref10] Brockmann H. Delhey J. Welzel C. Yuan H. (2009). The China puzzle: falling happiness in a rising economy. J. Happiness Stud. 10, 387–405. doi: 10.1007/s10902-008-9095-4

[ref11] Cava M. A. Fay K. E. Beanlands H. J. McCay A. E. Wignall R. (2005). Risk perception and compliance with quarantine during the SARS outbreak. J. Nurs. Scholarsh. 37, 343–347. doi: 10.1111/j.1547-5069.2005.00059.x, PMID: 16396407

[ref12] Chan J. F. W. Yuan S. Kok K. H. To K. K. W. Chu H. Yang J.. (2020). A familial cluster of pneumonia associated with the 2019 novel coronavirus indicating person-to-person transmission: a study of a family cluster. Lancet 395, 514–523. doi: 10.1016/S0140-6736(20)30154-9, PMID: 31986261 PMC7159286

[ref13] Dai B. Fu D. Meng G. Liu B. Liu X. (2020). The effects of governmental and individual predictors on covid protective behaviors in China: a path analysis model. Public Admin. Rev. 80, 797–804. doi: 10.1111/puar.13236, PMID: 32836438 PMC7276878

[ref14] De Pelsmacker P. Driesen L. Rayp G. (2005). Do consumers care about ethics? Willingness to pay for fair trade coffee. J. Consum. Aff. 39, 363–385. doi: 10.1111/j.1745-6606.2005.00019.x

[ref15] Durham D. P. Casman E. A. Albert S. M. (2012). Deriving behavior model parameters from survey data: self-protective behavior adoption during the 2009–2010 influenza a(H1N1) pandemic. Risk Anal. 32, 2020–2031. doi: 10.1111/j.1539-6924.2012.01823.x, PMID: 22563796 PMC3755610

[ref16] El-Said O. Aziz H. (2021). Virtual tours a means to an end: an analysis of virtual tours’ role in tourism recovery post COVID-19. J. Travel Res. 61, 528–548. doi: 10.1177/0047287521997567

[ref17] Feng T. Keller L. R. Wu P. Xu Y. (2014). An empirical study of the toxic capsule crisis in China: risk perceptions and behavioral responses. Risk Anal. 34, 698–710. doi: 10.1111/risa.12099, PMID: 23859541

[ref18] Ferrer-I-Carbonell A. Frijters P. (2004). How important is methodology for the estimates of the determinants of happiness? Econ. J. 114, 641–659. doi: 10.1111/j.1468-0297.2004.00235.x, PMID: 36222624

[ref19] Floyd D. L. Steven P. D. Rogers R. W. (2000). A meta-analysis of research on protection motivation theory. J. Appl. Soc. Psychol. 30, 407–429. doi: 10.1111/j.1559-1816.2000.tb02323.x, PMID: 36383540

[ref20] Fukukawa K. (2003). A theoretical review of business and consumer ethics research: normative and descriptive approaches. Mark. Rev. 3, 381–401. doi: 10.1362/146934703771910035

[ref21] Gladwin C. H. Gladwin H. Peacock W. G. (2001). Modeling hurricane evacuation decisions with ethnographic methods. Int. J. Mass Emerg. Disast. 19, 117–143. doi: 10.1177/028072700101900201

[ref22] Hao N. Wang H. H. Zhou Q. (2020). The impact of online grocery shopping on stockpile behavior in Covid-19. China Agric. Econ. Rev. 12, 459–470. doi: 10.1108/CAER-04-2020-0064, PMID: 35145946

[ref23] Hirose Y. Sonehara N. (2008). Management of information-credibility risk in an ICT society: a social implementation. Internet Res. 18, 142–154. doi: 10.1108/10662240810862202

[ref24] Hu X. Zhang X. Wei J. (2019). Public attention to natural hazard warnings on social media in China. Weather Clim. Soc. 11, 183–197. doi: 10.1175/WCAS-D-17-0039.1

[ref25] Jiang S. Lu M. Sato H. (2012). Identity, inequality, and happiness: evidence from urban China. World Dev. 40, 1190–1200. doi: 10.1016/j.worlddev.2011.11.002

[ref26] John K. Lina S. Ramani G. (2009). Subjective well-being and its determinants in rural China. China Econ. Rev. 20, 635–649. doi: 10.1016/j.chieco.2008.09.003

[ref27] Johnson B. B. (2019). Americans’ views of voluntary protective actions against zika infection: conceptual and measurement issues. Risk Anal. 39, 2694–2717. doi: 10.1111/risa.13378, PMID: 31339584

[ref28] Johnson B. B. Mayorga M. (2021). Americans’ early behavioral responses to COVID-19. Hum. Ecol. Risk. Assess. 27, 1733–1746. doi: 10.1080/10807039.2021.1884842

[ref29] Klemm C. Das E. Hartmann T. (2016). Swine flu and hype: a systematic review of media dramatization of the h1n1 influenza pandemic. J. Risk Res. 19, 1–20. doi: 10.1080/13669877.2014.923029

[ref30] Leung C. C. Lam T. H. Cheng K. K. (2020). Mass masking in the COVID-19 epidemic: people need guidance. Lancet 395:945. doi: 10.1016/S0140-6736(20)30520-1, PMID: 32142626 PMC7133583

[ref31] Li Q. Guan X. Wu P. Wang X. Y. Zhou L. Tong Y. Q.. (2020). Early transmission dynamics in Wuhan, China, of novel coronavirus-infected pneumonia. New. Engl. J. Med. 328, 1199–1207. doi: 10.1056/NEJMoa2001316, PMID: 31995857 PMC7121484

[ref32] Li J. Hallsworth A. G. Stefaniak J. A. (2020). Changing grocery shopping behaviors among chinese consumers at the outset of the covid-19 outbreak. Tijdschr. Econ. Soc. Genet. 111, 574–583. doi: 10.1111/tesg.12420, PMID: 32836486 PMC7307130

[ref33] Lindell M. K. Arlikatti S. Prater C. S. (2009). Why people do what they do to protect against earthquake risk: perceptions of hazard adjustment attributes. Risk Anal. 29, 1072–1088. doi: 10.1111/j.1539-6924.2009.01243.x, PMID: 19508448

[ref34] Lindell M. K. Huang S. K. Wei H. L. Samuelson C. D. (2016). Perceptions and expected immediate reactions to tornado warning polygons. Nat. Hazards 80, 683–707. doi: 10.1007/s11069-015-1990-5, PMID: 29119587

[ref35] Lindell M. K. Perry R. W.. Behavioral foundations of community emergency planning, Washington: DC: Hemisphere Press (1992).

[ref36] Lindell M. K. Perry R. W. Communicating environmental risk in multiethnic communities. Thousand Oaks, CA: Sage (2004).

[ref37] Lindell M. K. Perry R. W. (2012). The protective action decision model: theoretical modifications and additional evidence. Risk Anal. 32, 616–632. doi: 10.1111/j.1539-6924.2011.01647.x, PMID: 21689129

[ref38] Liu S. Huang J. C. Brown G. L. (1998). Information and risk perception: a dynamic adjustment process. Risk Anal. 18, 689–699. doi: 10.1111/j.1539-6924.1998.tb01113.x, PMID: 9972578

[ref39] McComas K. A. (2006). Defining moments in risk communication research: 1996–2005. J. Health Commun. 11, 75–91. doi: 10.1080/10810730500461091, PMID: 16546920

[ref40] Moreb N. A. Albandary A. Jaiswal S. Jaiswal A. K. (2021). Fruits and vegetables in the management of underlying conditions for COVID-19 high-risk groups. Foods 10:389. doi: 10.3390/foods10020389, PMID: 33578926 PMC7916708

[ref41] Myae A. C. Goddard E. (2020). Household behavior with respect to meat consumption in the presence of BSE and CWD. Can. J. Agric. Econ. 68, 315–341. doi: 10.1111/cjag.12223

[ref42] Peltz R. Avisar-Shohat G. Bar-Dayan Y. (2007). Differences in public emotions, interest, sense of knowledge and compliance between the affected area and the nationwide general population during the first phase of a bird flu outbreak in Israel. J Infect. 55, 545–550. doi: 10.1016/j.jinf.2007.07.014, PMID: 17826838

[ref43] Pennycook G. Mcphetres J. Zhang Y. Lu J. G. Rand D. G. (2020). Fighting covid-19 misinformation on social media: experimental evidence for a scalable accuracy-nudge intervention. Psychol. Sci. 31, 770–780. doi: 10.1177/0956797620939054, PMID: 32603243 PMC7366427

[ref44] Poland G. A. (2010). The 2009–2010 influenza pandemic: effects on pandemic and seasonal vaccine uptake and lessons learned for seasonal vaccination campaigns. Vaccine 28, D3–D13. doi: 10.1016/j.vaccine.2010.08.024, PMID: 20713258

[ref45] Prentice C. Quach S. Thaichon P. (2020). Antecedents and consequences of panic buying: the case of COVID. Int. J. Consum. Stud. 46, 132–146. doi: 10.1111/ijcs.12649

[ref46] Prochaska J. O. Velicer W. F. (1997). The transtheoretical model. Am. J. Health Promot. 12, 6–7. doi: 10.4278/0890-1171-12.1.6, PMID: 10170434

[ref47] Qazi A. Qazi J. Naseer K. Zeeshan M. Hardaker G. Maitama J. Z.. (2020). Analyzing situational awareness through public opinion to predict adoption of social distancing amid pandemic covid-19. J. Med. Virol. 92, 849–855. doi: 10.1002/jmv.25840, PMID: 32266990 PMC7262187

[ref48] Ren Y. Li H. Wang X. (2019). Family income and nutrition-related health: evidence from food consumption in China. Soc. Sci. Med. 232, 58–76. doi: 10.1016/j.socscimed.2019.04.016, PMID: 31071477

[ref49] Rogers R. W. (1983). Cognitive and physiological processes in fear appeals and attitude change: a revised theory of protection motivation. Soc. Psychophysiol. 19, 469–479. doi: 10.1016/0022-1031(83)90023-9

[ref50] Sapp S. G. Downing-Matibag T. (2009). Consumer acceptance of food irradiation: a test of the recreancy theorem. Int. J. Consum. Stud. 33, 417–424. doi: 10.1111/j.1470-6431.2009.00772.x

[ref51] Scacchi A. Catozzi D. Boietti E. Bert F. Siliquini R. (2021). COVID-19 lockdown and self-perceived changes of food choice, waste, impulse buying and their determinants in Italy: quaranteat, a cross-sectional study. Foods 10:306. doi: 10.3390/foods10020306, PMID: 33540825 PMC7913081

[ref52] Shapira S. Aharonson-Daniel L. Bar-Dayan Y. (2018). Anticipated behavioral response patterns to an earthquake: the role of personal and household characteristics, risk perception, previous experience and preparedness. Int. J. Disast. Risk Res. 31, 1–8. doi: 10.1016/j.ijdrr.2018.04.001

[ref53] Shi M. Cheng X. Zhang X. H. (2020). Impacts of the COVID-19 pandemic on consumers' food safety knowledge and behavior in China. J. Integr. Agric. 19, 2926–2936. doi: 10.1016/S2095-3119(20)63388-3, PMID: 35755618 PMC9215339

[ref54] Shi G. Q. Zhong X. N. He W. Liu H. Liu X. Y. Ma M. Z. (2021). Factors influencing protective behavior in the post-covid-19 period in China: a cross-sectional study. Environ. Health Prev. 26:95. doi: 10.1186/s12199-021-01015-2, PMID: 34556043 PMC8459581

[ref55] Slater M. D. Rasinski K. A. (2005). Media exposure and attention as mediating variables influencing social risk judgments. J. Commun. 55, 810–827. doi: 10.1111/j.1460-2466.2005.tb03024.x

[ref56] Terpstra T. Lindell M. K. (2013). Citizens’ perceptions of flood hazard adjustments an application of the protective action decision model. Environ. Behav. 45, 993–1018. doi: 10.1177/0013916512452427

[ref57] Thomas M. S. Feng Y. (2021). Consumer risk perception and trusted sources of food safety information during the covid-19 pandemic. Food Control 130:108279. doi: 10.1016/j.foodcont.2021.108279, PMID: 36568483 PMC9759357

[ref58] Trumbo C. W. Mccomas K. A. (2003). The function of credibility in information processing for risk perception. Risk Anal. 23, 343–353. doi: 10.1111/1539-6924.00313, PMID: 12731818

[ref59] Vong S. O'Leary M. Feng Z. (2014). Early response to the emergence of influenza a(H7N9) virus in humans in China: the central role of prompt information sharing and public communication. Br. World Health Organ. 92, 303–308. doi: 10.2471/BLT.13.125989, PMID: 24700999 PMC3967572

[ref60] Vuoric V. Okkonen J. (2012). Knowledge sharing motivational factors of using an intra-organizational social media platform. J. Knowl. Manag. 16, 592–603. doi: 10.1108/13673271211246167

[ref61] Wang E. An N. Gao Z. F. Kiprop E. Geng X. H. (2020). Consumer food stockpiling behavior and willingness to pay for food reserves in COVID-19. Food Secur. 12, 739–747. doi: 10.1007/s12571-020-01092-1, PMID: 32837661 PMC7406878

[ref62] Wang F. Wei J. C. Huang S. K. Lindell M. K. Ge Y. Wei H. L. (2018). Public reactions to the 2013 Chinese H7N9 influenza outbreak: perceptions of risk, stakeholders, and protective actions. J. Risk Res. 21, 809–833. doi: 10.1080/13669877.2016.1247377

[ref63] Wang F. Wei J. C. Shi X. (2018). Compliance with recommended protective actions during an H7N9 emergency: a risk perception perspective. Disasters 42, 207–232. doi: 10.1111/disa.12240, PMID: 28799670

[ref64] Wei J. Bing B. Liang L. (2012). Estimating the diffusion models of crisis information in micro blog. J. Inform. 6, 600–610. doi: 10.1016/j.joi.2012.06.005

[ref65] Wei J. C. Ouyang Z. Chen H. P. (2017). Well known or well liked? The effects of corporate reputation on firm value at the onset of a corporate crisis. Strateg. Manage J. 38, 2103–2120. doi: 10.1002/smj.2639, PMID: 36813977

[ref66] Wei J. Wang F. Lindell M. K. (2016). The evolution of stakeholders’ perceptions of disaster: a model of information flow. J. Assoc. Inform. Sci. Technol. 67, 441–453. doi: 10.1002/asi.23386

[ref67] Wei J. Wang F. Zhao D. (2012). A risk perception model: simulating public response to news reports in China. Inf. Res. 21, 411–436. doi: 10.1007/s00778-011-0251-9

[ref68] Wei J. Zhao D. Yang F. Du S. Marinova D. (2010). Timing crisis information release via television. Disasters 34, 1013–1030. doi: 10.1111/j.1467-7717.2010.01180.x, PMID: 20572851

[ref69] Weinstein N. D. (1988). The precaution adoption process. Health Psychol. 7, 355–386. doi: 10.1037/0278-6133.7.4.355, PMID: 3049068

[ref70] Xie X. Huang L. Li J. J. Zhu H. (2020). Generational differences in perceptions of food health/risk and attitudes toward organic food and game meat: the case of the covid-19 crisis in China. Int. J. Environ. Res. Pub. Health 17:3148. doi: 10.3390/ijerph17093148, PMID: 32366016 PMC7246561

[ref71] Youn S. Y. Rana M. R. I. Kopot C. (2022). Consumers going online for big-box retailers: exploring the role of feeling disconnected during a pandemic. Int. J. Consum. Stud. 46, 2383–2403. doi: 10.1111/ijcs.12793

[ref72] Zavyalova A. Pfarrer M. D. Reger R. K. Shapiro D. L. (2012). Managing the message: the effects of firm actions and industry spillovers on media coverage following wrongdoing. Acad. Manag. J. 55, 1079–1101. doi: 10.5465/amj.2010.0608

[ref73] Zheng L. Elhai J. D. Miao M. Wang Y. Wang Y. Gan Y. (2022). Health-related fake news during the COVID-19 pandemic: perceived trust and information search. Int. Res. 32, 768–789. doi: 10.1108/INTR-11-2020-0624

[ref74] Zhu D. Xie X. Gan Y. (2011). Information source and valence: how information credibility influences earthquake risk perception. J. Environ. Psychol. 31, 129–136. doi: 10.1016/j.jenvp.2010.09.005

[ref75] Zhu W. W. Yao N. Z. (2018). Public risk perception and intention to take actions on city smog in China. Hum. Ecol. Risk. Assess. 25, 1531–1546. doi: 10.1080/10807039.2018.1471340

